# Circulating Long Noncoding RNA UCA1 as a Novel Biomarker of Acute Myocardial Infarction

**DOI:** 10.1155/2016/8079372

**Published:** 2016-02-01

**Authors:** Youyou Yan, Bin Zhang, Ning Liu, Chao Qi, Yanlong Xiao, Xin Tian, Tianyi Li, Bin Liu

**Affiliations:** Department of Cardiology, Second Hospital of Jilin University, No. 218 Ziqiang Street, Changchun 130041, China

## Abstract

Acute myocardial infarction (AMI) is the most serious cardiovascular disease with high morbidity and mortality. Recent studies have showed that long noncoding RNAs (lnc RNA) play important roles in pathophysiology of cardiovascular diseases, but the investigations are still in their infancy. An lnc RNA named urothelial carcinoma-associated 1 (UCA1) is found in tumors such as bladder cancers and lung cancer. And the UCA1 could be as a predictive biomarker for bladder cancer in urine samples or lung cancer in plasma, respectively. In normal states, UCA1 is specifically expressed in heart of adult, indicating that UCA1 might be as a biomarker for heart diseases such as AMI. To test the speculation, we detect the level of UCA1 in plasma of AMI patients and health control using quantitative reverse transcription-polymerase chain reaction (qRT-PCR). In addition, we also test the level of miR-1 as it is reported to regulate the expression of UCA1. The results show that the level of plasma UCA1 is decreased at the early state of AMI patients and increased at day 3 after AMI. In addition, the UCA1 alteration is inversely associated with the expression of miR-1. These findings indicate that the circulating UCA1 could be used as a promising novel biomarker for the diagnosis and/or prognosis of AMI.

## 1. Introduction

Acute myocardial infarction (AMI) is the most serious cardiovascular disease with high morbidity and mortality [1]. Rapid and correct diagnosis of AMI has been a focus of research as it could prevent the progressive development of AMI and improve the cure and survival rate in patients [2]. Cardiac troponins are the most common biomarkers used for diagnosis of AMI in clinical practice. However, there are several conditions other than acute myocardial infarction which could result in elevated cardiac troponin levels. Therefore, there is still a clinical need for novel biomarker [3].

Increasing evidences suggest that noncoding RNAs (nc RNAs) including microRNAs (miRNAs) and long nc RNAs (lnc RNAs) play important roles in pathophysiology of cardiovascular diseases including AMI [4]. Of them, microRNAs (miR) with 21–25 nucleotides in length have been largely investigated in cardiovascular disease. For example, miR-1, miR-133, miR-499, and miR-208 are increased in serum of patients with AMI [5–8]. Recently, lnc RNAs with longer than 200 nucleotides in length also were found to regulate gene expression by diverse mechanisms [9]. In cardiovascular disease states, the profiles of lnc RNAs in plasma and serum have been found to be altered, suggesting broad opportunities for development of circulating lnc RNAs as blood-based markers for molecular diagnostics. For example, the circulating long noncoding RNA LIPCAR was explored as prognostic biomarkers for heart failure [10]. In addition, several lnc RNAs have been found to be regulated in the heart tissue during AMI [11]. However, studies about the circulating long noncoding RNA as biomarker of AMI are still in their infancy. To date, only two studies have analyzed altered circulating lnc RNAs in patients with myocardial infarction.

An ideal biomarker for AMI required high sensitivity and specificity as well as good accessibility and predictability [12]. An lnc RNA named urothelial carcinoma-associated 1 (UCA1) might meet the criteria. The UCA1 originally was identified in human bladder transitional cell carcinoma and highly expressed in lung cancer. In normal states, UCA1 is only expressed in heart and spleen after birth and higher expression in heart [13, 14], indicating that UCA1 might be as a specific biomarker for heart. Besides the specificity, the UCA1 could be detected in freshly voided urine samples or plasma and be as a predictive biomarker for bladder cancer or lung cancer, respectively [13, 15]. In the early stages of AMI, pathological changes such as myocardial ischemia, hypoxia, edema, and necrosis occur rapidly, followed by the release of necrotic products, such as cardiac troponins (cTns), creatine kinase (CK), and brain natriuretic peptide (BNP), into the bloodstream [16]. The lnc RNA might be released from necrotic products and detected in the plasma, indicating UCA1 might be easily obtained. In addition, recent study shows that the UCA1 influences the cell proliferation, apoptosis, and cell cycle distribution of colorectal cancer [17]. Furthermore, UCA1 could protect cardiomyocyte against H_2_O_2_-induced cardiomyocyte apoptosis [18]. Whereas hypoxia or reoxygenation-induced cardiomyocyte apoptosis is one of the major causes of AMI, indicating that UCA1 might be as the predictive biomarker for the diagnosis and/or prognosis of AMI.

The objectives of the study were to test whether UCA1 could be as the biomarker of AMI. We tested the level of UCA1 in AMI and non-AMI subjects using qRT-PCR and compare its diagnostic value with that of cTnI and CK-MB.

## 2. Method and Material

### 2.1. Participants

Between 2014 November and 2015 January, we studied 49 AMI patients at ages of 30–75 and 15 non-AMI subjects at ages of 37–63 from the Second Hospital of Jilin University (Changchun, China). AMI was diagnosed based on combination of several parameters: ischemic symptom plus increased cardiac troponin I (cTnI) and creatine kinase-MB (CK-MB), pathological Q wave. ST-segment elevation or depression was defined by the European Society of Cardiology/American College of Cardiology. Baseline ECG was recorded in all patients. Written consents were obtained from all subjects studied and the study protocol was approved by the ethics committee of the Jilin University.

### 2.2. Isolation of Human Plasma

For lnc RNA and miRNA detection, whole blood (WB) samples (5 mL per patient) were collected from subjects via a direct venous puncture into tubes containing sodium citrate, centrifuged at 1000 g for 5 min, and then the supernatant (plasma) was carefully transferred into an RNase-free tube for extraction of RNA.

### 2.3. RNA Isolation and Real-Time Quantitative RT-PCR (qRT-PCR)

For measuring microRNAs, miRNA was extracted from the plasma samples using the miRcute miRNA Isolation kit (TIAN GEN, Applied Biosystem). The samples performed both poly-(A) tailing and reverse transcription with the miScript reverse transcription kit (TIAN GEN). miR-1 was quantitated by using TaqMan miRNA quantitative reverse transcriptase-polymerase chain reaction (qRT-PCR) assay according to the protocol of the manufacturer. U6 RNA was performed as a miRNA internal control.

For measuring lnc RNAs, 1 *μ*g total RNA was reverse transcribed using MulV reverse transcriptase (Transgen, Cat. AT101-02) and random hexamer primers in a 20 *μ*L reaction. The SYBR Green PCR Master Mix Kit (Transgen, Cat. AQ131-01) was used in real-time PCR for relative quantification of miRNAs and of UCA1; in this study U6 and 18S were used as an internal control, respectively. qRT-PCR was performed on 7500 FAST Real-Time PCR System (Applied Biosystems). The RT primers used were (1) miR-1 forward: GGGGTGGAATGTAAAGAA and miR-1 reversed: TGCGTGTCGTGGAGTC; (2) U6 forward: GCTTCGGCAGCACATATACTAAAAT and U6 reversed: CGCTTCACGAATTTGCGTGTCAT; (3) UCA1 forward: ACGCTAACTGGCACCTTGTT and UCA1 reversed: TGGGGATTACTGGGGTAGGG; (4) 18S forward: CAGCCACCCGAGATTGAGCA and 18S reversed: TAGTAGCGACGGGCGGTGTG.

Analysis of relative gene expression levels was performed using the formula 2^−ΔCT^ with ΔCT = CT_(target gene)_ − CT_(control)_.

### 2.4. Statistical Analysis

Data were described as means ± SD and median for general characteristics of subjects. All statistical analyses were performed using the SPSS16.0 software. Differences between different groups were assessed using One-Way ANOVA comparison method. Value of *P* < 0.05 was considered to indicate statistical significance. Receiver operating characteristic (ROC) curves were established to evaluate the predictive power of circulating UCA1 between the AMI and non-AMI subjects. The area under the ROC curve (AUC) was used to assess the predictive power. The sensitivity and specificity were calculated according to the standard formulas.

## 3. Results

### 3.1. The Baseline Clinical Characteristics of the Study Subjects

A total of 49 AMI patients and 15 non-AMI subjects were studied to determine the association of circulating UCA1 levels with AMI. The baseline clinical characteristics of the study subjects are shown in Table 1. There were significant differences in cTnI and CK-MB between AMI and non-AMI subjects (*P* < 0.01, *P* < 0.01, resp.). There were no statistical differences in age, HDL, LDL, EF%, hypertension, and history of diabetes and smoking status between the AMI and non-AMI subjects.

### 3.2. The Level of Circulating UCA1 in the Patients with AMI

**Figure 1:**
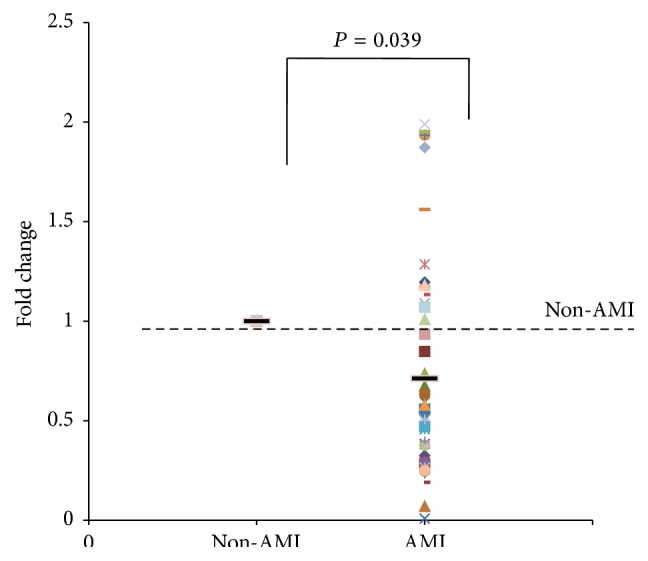
The level of UCA1 in plasma of AMI patients. The expression levels of UCA1 in plasma of hospitalized AMI patients (*n* = 49) and non-AMI subjects (*n* = 15) were analyzed by qRT-PCR. The results are shown as the means, *P*, versus non-AMI subjects.

To investigate whether UCA1 could be as the novel biomarker for the diagnosis of AMI, its expression levels in plasma of AMI patients and health subjects were analyzed. In the non-AMI subjects, the level of circulating UCA1 was very low and the mean CT value was 28.43 by 45 cycles of Q-PCR. The results (Figure 1) showed that the expression of UCA1 was significantly decreased in plasma of patients with AMI, compared with non-AMI subjects (*P* < 0.05). However, we also found the levels of UCA1 were increased in some samples. We speculated that the level of circulating UCA1 in patients with AMI might be correlated with the time after onset of AMI.

**Figure 2:**
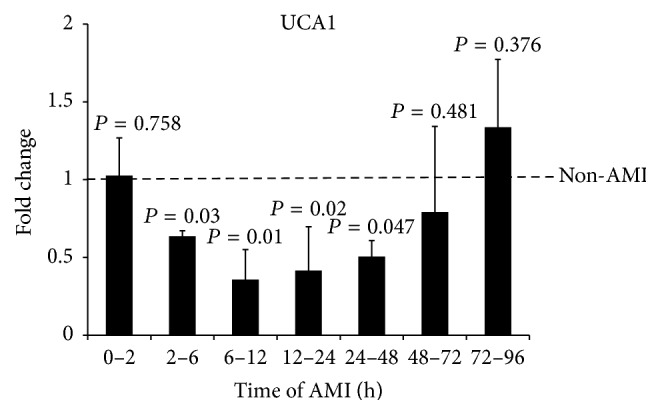
The levels of circulating UCA1 in different time of onset of AMI. The expression level UCA1 in plasma of AMI patients (*n* = 49) were analyzed by qRT-PCR according to the time after onset of AMI. The results are shown as the means, *P*, versus non-AMI subjects.

To test the speculation, the patients with AMI were divided into 7 groups according to the time after AMI: 0–2 hours (2 cases), 2–6 hours (3 cases), 6–12 hours (6 cases), 12–24 hours (12 cases), 24–48 hours (13 cases), 48–72 hours (9 cases), and 72–96 hours (4 cases). As shown in Figure 2, the levels of UCA1 in AMI patients were decreased in 2–6 h, 6–12 h, 12–24 h, and 24–48 h after AMI (*P* < 0.05). The level of UCA1 was decreased from 2 h after AMI and was lowest in 6–12 h. Then, the level of circulating UCA1 began to recover and back to the control value in 48–72 h after onset of AMI. The level of UCA1 in 72–96 h after onset of AMI was higher than non-AMI subjects.

### 3.3. The Level of Circulating miR-1 in the Patients with AMI

**Figure 3:**
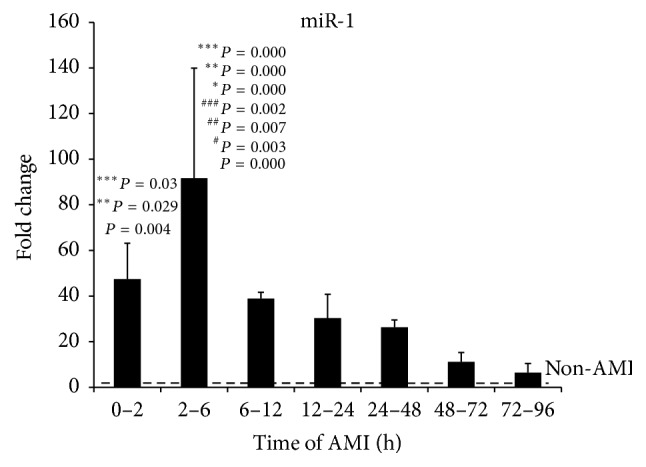
The levels of circulating miR-1 in different time of onset of AMI. The expression level of miR-1 in plasma of AMI patients (*n* = 49) were analyzed by qRT-PCR according to the time after onset of AMI. The results were shown as the means, *P*, versus non-AMI subjects; ^#^ *P*, versus 0–2 h; ^##^ *P*, versus 6–12 h; ^###^ *P*, versus 12–24 h; ^*∗*^ *P*, versus 24–48 h; ^*∗∗*^ *P*, versus 48–72 h; ^*∗∗∗*^ *P*, versus 72–96 h.

It was reported that miR-1 inhibited the expression of UCA1 [19]. Therefore, we investigate whether the expression levels of UCA1 are associated with miR-1. The level of plasma miR-1 was also tested according to the time after AMI. The results (Figure 3) showed that the level of miR-1 was increased from 0 to 2 h, compared with health control (*P* < 0.05), and was highest in 2–6 h. Then, it began to decrease and returned to the normal level at 72–96 h. There was statistical significance between the patients at 2–6 h after onset of AMI and health subjects or other patients (*P* < 0.05). The results showed that the expression of UCA1 was negatively correlated with the expression of miR-1.

### 3.4. The Effect of Hypertension and Diabetes on Circulating UCA1

**Figure 4:**
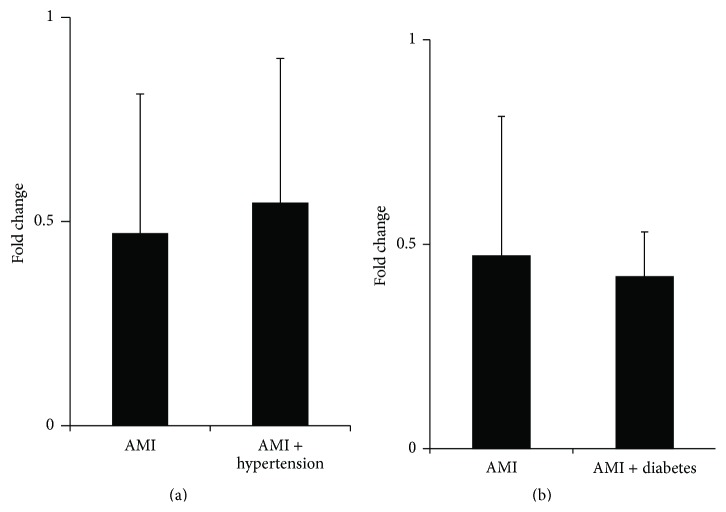
The influence of hypertension and diabetes on level of UCA1 in plasma of AMI patients. The expression level UCA1 in plasma of AMI patients (*n* = 34) at 2–48 h was analyzed by qRT-PCR according to the patients with or without hypertension or diabetes. (a) AMI and AMI combined hypertension subjects. (b) AMI and AMI combined diabetes subjects. The results are shown as the means, *P*, versus non-AMI subjects.

Many AMI patients have the history of hypertension and diabetes. We further investigated the influence of hypertension and diabetes on the level of UCA1. As the level of UCA1 was lowest at 6–48 h after AMI, those patients were divided into AMI alone group, AMI + hypertension group, and AMI + diabetes group and the level of UCA1 in different groups was analyzed. The results (Figure 4(a)) showed that there were no significant differences in the UCA1 expression between AMI sand AMI combined with hypertension subjects. Similarly, there were no significant differences in the UCA1 expression between AMI and AMI combined with diabetes (Figure 4(b)). Those results indicated that the UCA1 was not correlated with hypertension or diabetes.

### 3.5. The Predictive Power of UCA1 for AMI

**Figure 5:**
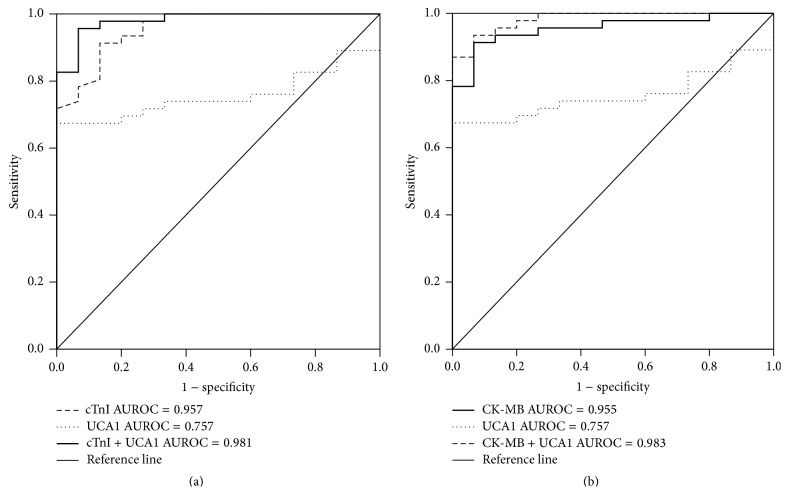
Comparisons of the sensitivity and specificity of the diagnosis by plasma UCA1 and cTnT in the AMI patients. ROC curves were constructed to evaluate the diagnostic values of UCA1 for the AMI patients in comparison with cTnI and CK-MB. (a) Comparison of UCA1 and nTnI. (b) Comparison of UCA1 and CK-MB. AUC: area under the ROC curve.

To evaluate the predictive power of circulating UCA1 for AMI, we performed ROC analysis for 49 patients with AMI. As shown in Figure 5(a), the area under the ROC curve (AUC) for UCA1 was 0.757 (95% confidence interval) and the AUC measured for cTnI was 0.957, while the AUC measured for UCA1-cTnI was 0.981. As shown in Figure 5(b), the area under the ROC curve (AUC) for UCA1 was 0.757 (95% confidence interval) and the AUC measured for CK-MB was 0.9592, while the AUC measured for UCA1-CK-MB was 0.983. This result demonstrated that UCA1 had marked sensitivity and specificity for AMI, but it was not superior to cTnI and CK-MB for the diagnosis of AMI.

## 4. Discussion

In this study, we investigated whether plasma lnc RNA UCA1 could be as biomarker of AMI. Interestingly, the levels of plasma UCA1 were decreased at the early state of AMI patients and increased at day 3 after AMI, which was not affected in AMI patient with hypertension or diabetes. Moreover, the level of UCA1 was negatively correlated with the expression of miR-1.

The UCA1 played important role on embryonic development and only was expressed in heart and spleen of normal adult after birth [14]. The UCA1 might play important role in protecting heart. UCA1 promoted glucose consumption and lactate production in bladder cancer cells [20], indicating that the UCA1 might be important in providing the energy needed for heart. In addition to involvement in glucose metabolic processes, UCA1 also could promote cell proliferation and inhibit cell apoptosis in bladder cancer [21]. It was also supported that UCA1 could inhibit the expression of p27 [22], while upregulation of p27 significantly enhanced cell apoptosis [23]. Consistently, it was found that UCA1 inhibited the expression of miR-1 and protected the cardiomyocyte against H_2_O_2_-induced apoptosis [24]. Our study showed that the circulating UCA1 was decreased in the plasma of patients with AMI, indicating that the cardiomyocyte apoptosis in AMI might be associated with downregulation of UCA1.

Interestingly, the UCA1 was decreased early but upregulated afterwards in the plasma of patients after myocardial infarction. Similarly, global transcriptomic analyses uncovered that LIPCAR (the mitochondrial long noncoding RNA uc022bqs.1) was downregulated early but upregulated afterwards in the plasma of patients after myocardial infarction [10]. The level of UCA1 was regulated by miR-1. It was found that miR-1 decreased the expression of UCA1 in bladder cancer cells in an Ago2-slicer-dependent manner [19]. In addition, the binding site of miR-1 and UCA1 was also found, indicating that miR-1 could directly inhibit the expression of UCA1 [19]. The miR-1 was markedly increased in the plasma of the rats or patients with AMI [25]. Our study also showed that the UCA1 was negatively correlated with the expression of miR-1. The UCA1 was decreased at the early state of AMI and increased at day 3 after AMI, while the miR-1 was increased in the early state of AMI and returned to the normal level on day 3 after the onset of AMI. The miR-1 was found to exacerbate cardiac injury by affecting the expression of a host of protective proteins such as BCL2 and HSP60 [26] and also by inhibition of UCA1. In addition, some microRNAs are selectively deplenished from plasma during their passage through the myocardial circulation. For example, circulating levels of miR-126 decreased during transcoronary passage in patients with evidence of myocardial injury, suggesting consumption during transcoronary passage. Those microRNAs might be consummated by some circulating lnc RNAs [27].

*Limitations*. It should be noted that the consideration of circulating lnc RNA (UCA1) as a biomarker for AMI is at present based on our results from a relatively small sample size and larger clinical studies are definitely required to establish the case. UCA1 had marked sensitivity and specificity for AMI, but it was not superior to cTnI and CK-MB for the diagnosis of AMI. However, UCA1 as an additional factor might enhance the clinical use of cTnI and CK-MB. While the fact that circulating was restored back to normal in AMI patients on discharge may give an insinuation for the potential of a prognostic marker as well. In addition, it should be concerned that the UCA1 was highly expressed in many cancers such as bladder cancer and lung cancer and could be as predictor biomarker for those cancers. Furthermore, UCA1 is also expressed in the spleen in normal states, indicating that the level might be changed with immune dysfunction. Therefore, those conditions must be excluded when UCA1 was used as biomarker for AMI. Application of detection technology for lnc RNA is challenged. Although real-time PCR is the gold standard for gene expression quantification and accepted as being a powerful technique in comparative expression analysis in life sciences and medicine [19], it was very expensive and time-consuming for a large quantity of samples. Last, the emerging application of circulating lnc RNAs for disease diagnosis is restrained by the limited knowledge that we have of their biology. For instance, it remains unclear whether lnc RNAs contribute to the disease or if they become altered as a consequence of the disease itself.

Taken together, our data demonstrates that UCA1 is decreased in the early state of AMI and negatively correlated with miR-1, suggesting that UCA1 participates in the pathophysiology of AMI interaction with miR-1. Although UCA1 as independent biomarker displays sensitivity and specificity compared to cTnI and CK-MB, it could be as additional biomarker to enhance sensitivity and specificity than cTnI and CK-MB. It is unquestionable that the more we learn about lnc RNA expression patterns in AMI disease, the higher the chances for an improved diagnosis and better prognosis will be [28].

## Figures and Tables

**Table 1:** Clinical characteristics of patients.

Characteristics	AMI (*n* = 49)	Non-AMI (*n* = 15)	*P*
Age (years)	61.02 ± 11.83	58.13 ± 10.18	0.42
Male/female (*n*/*n*)	30/19	7/8	0.75
HDL C (mmol/L)	1.05 ± 0.35	1.28 ± 0.23	0.52
Current smoking, *n* (%)	25 (51%)	5 (33%)	0.161
EF%	55.02 ± 8.72	69.54 ± 6.78	0.72
LDL C (mmol/L)	2.76 ± 0.76	2.13 ± 0.84	0.68
Diabetes, *n* (%)	12 (24.5%)	4 (26.7%)	0.938
CK-MB (U/L)	74.35 ± 52.04	3.05 ± 1.47	<0.01
cTnI (ng/mL)	5.34 ± 3.57	0.032 ± 0.022	<0.01
Hypertension, *n* (%)	31 (63.2%)	6 (53.3%)	0.324
